# Non-Coding Changes Cause Sex-Specific Wing Size Differences between Closely Related Species of *Nasonia*

**DOI:** 10.1371/journal.pgen.1000821

**Published:** 2010-01-15

**Authors:** David W. Loehlin, Deodoro C. S. G. Oliveira, Rachel Edwards, Jonathan D. Giebel, Michael E. Clark, M. Victoria Cattani, Louis van de Zande, Eveline C. Verhulst, Leo W. Beukeboom, Monica Muñoz-Torres, John H. Werren

**Affiliations:** 1 Department of Biology, University of Rochester, Rochester, New York, United States of America; 2 Evolutionary Genetics, Centre for Ecological and Evolutionary Studies, University of Groningen, Haren, The Netherlands; 3 Clemson University Genomics Institute, Clemson University, Clemson, South Carolina, United States of America; Princeton University, Howard Hughes Medical Institute, United States of America

## Abstract

The genetic basis of morphological differences among species is still poorly understood. We investigated the genetic basis of sex-specific differences in wing size between two closely related species of *Nasonia* by positional cloning a major male-specific locus, *wing-size1* (*ws1*). Male wing size increases by 45% through cell size and cell number changes when the *ws1* allele from *N. giraulti* is backcrossed into a *N. vitripennis* genetic background. A positional cloning approach was used to fine-scale map the *ws1* locus to a 13.5 kilobase region. This region falls between *prospero* (a transcription factor involved in neurogenesis) and the master sex-determining gene *doublesex*. It contains the 5′-UTR and *cis-*regulatory domain of *doublesex*, and no coding sequence. Wing size reduction correlates with an increase in *doublesex* expression level that is specific to developing male wings. Our results indicate that non-coding changes are responsible for recent divergence in sex-specific morphology between two closely related species. We have not yet resolved whether wing size evolution at the *ws1* locus is caused by regulatory alterations of *dsx* or *prospero,* or by another mechanism. This study demonstrates the feasibility of efficient positional cloning of quantitative trait loci (QTL) involved in a broad array of phenotypic differences among *Nasonia* species.

## Author Summary

The regulation of cell size and cell numbers is an important part of determining the size of organs in development, as well as of controlling cell over-proliferation in diseases such as cancer and diabetes. How the regulation of cell size and number can change to produce different organ sizes is not well understood. Here, we investigate the recent evolution of sex-specific wing size differences between two species that involve changes to cell size and number regulation. Males of the emerging genetic model wasp *Nasonia vitripennis* have small wings and do not fly, while males of the closely related species *N. giraulti* have large wings and do fly. We isolated a locus that contributes substantially to this wing size difference by increasing cell size and cell number. Surprisingly, we found that the determinant for this wing size difference is located in the non-coding region between two known transcription factors, the master sex determining gene *doublesex* and neurogenesis regulator *prospero*. The mechanism by which *ws1* regulates sex specific wing growth has yet to be determined, although differences in *dsx* expression level in developing male wings may indicate a role for this sex determination locus.

## Introduction

Somatic sexual differentiation is an ancient feature of animals, yet sex differences in morphological traits can evolve rapidly. Because of this, between-species genetic analysis of recently evolved sexual differences has been proposed as a way of identifying the genes and genetic changes that underlie morphological diversification [1]. For example, Kopp et al. [2] have found that a sex-specific abdominal pigmentation difference that recently evolved between *Drosophila* species is caused by non-coding *cis-*regulatory changes in the *bric-a-brac* gene, changes which involve binding sites for conserved transcription factors *doublesex* and *ABD-B* [3]. The study of recently evolved sex differences can therefore reveal changes in tissue- and sex-specific gene regulatory networks. Nevertheless, there have been few studies investigating the genetic and molecular basis of the recent evolution of morphological differences between species, due in part to the difficulty of conducting genetic analyses in diverged species that are often reproductively incompatible.

An active debate concerns whether the evolution of differences between species are due primarily to *cis*-regulatory or protein coding changes (e.g., [4]–[8]). While protein-coding changes have been the focus of most historical studies of phenotypic evolution, it has been argued that changes to non-coding *cis*-regulatory elements may be more important, as they are crucial to the spatiotemporal control of gene expression in development and can change with potentially fewer pleiotropic effects on other processes [4],[5]. However, empirical support for this claim is limited, largely by the difficulty of determining the genetic basis of phenotypic changes to a fine enough level to distinguish between *cis* and protein-coding changes [6]. An additional issue concerns whether the standing genetic variation for phenotypes within populations represent the same spectrum of mutations that go to fixation and become involved in species differences in phenotype [7]. Therefore, additional genetic studies of phenotypic evolution in recently diverged species are needed to help reveal the processes by which new morphologies evolve and the relative roles of *cis*-regulatory versus protein-coding changes in morphological evolution.

Here we investigate the genetic basis of male-specific differences between two species of *Nasonia*, *N. vitripennis* and *N. giraulti*. *Nasonia* is a complex of four closely related parasitic wasp species that is rapidly emerging as a model for evolutionary and developmental genetics [9],[10]. *Nasonia* males are haploid, and therefore can be readily genotyped for visible and molecular markers regardless of marker dominance. Furthermore, unlike most organisms, *Nasonia* species can be made inter-fertile in the lab by removing bacterial symbionts (*Wolbachia*) that cause sperm-egg incompatibilities among the species [11],[12]. This permits movement of genes involved in phenotypic differences between the species by backcrossing [13]–[15]. Utilizing flanking visible and recessive lethal mutations and genetic and genomic tools in *Nasonia*, positional cloning of genes involved in species differences can then be accomplished [9].

**Figure 1:**
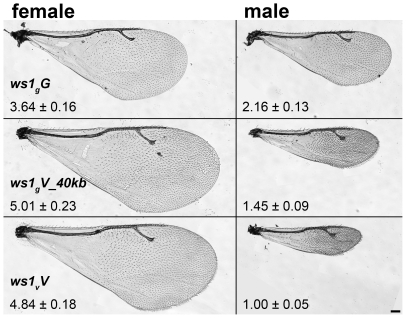
Wing size differences due to *ws1.* Wings of *N. giraulti* (*ws1_g_G*), *N. vitripennis* (*ws1_v_V*) and *giraulti ws1* in *vitripennis* background (*ws1_g_V_40kb*). Wing area ± S.D. relative to *ws1_v_V* males is shown (see also Table 1). Scale bar: 100 µm.

*N. giraulti* males have large wings (Figure 1) and are capable of flight, whereas *N. vitripennis* males have vestigial wings and do not fly, although they use them in courtship and agonistic displays [16]. A major portion of the male-specific wing-size difference is due to two loci, *wing-size1* (*ws1*) and *widerwing* (*wdw*) [13],[15]. Both *ws1* and *wdw* increase wing size in a sex specific fashion, as seen when introgressed from *N. giraulti* by backcrossing into an *N. vitripennis* background. In this study, we positionally clone the *ws1* locus to a 13.5 Kb non-coding region, which falls near the sex determining locus *doublesex* [17],[18] and includes its 5′ UTR. This is the first positional cloning of a gene in *Nasonia*, and the study illustrates methods for utilizing haplodiploidy for efficient cloning of interspecies QTL in this genetic system.

## Results/Discussion

### Sex-specific differences in wing size

*Nasonia* wings are composed of a larger forewing and smaller hindwing. Here we focus our attention on the forewing, although more subtle differences in the hindwing are also found between the species and sexes. *N. giraulti* male forewings are 2.16 fold larger in area than *N. vitripennis* male forewings, although female wings of both species are large and more similar in size (Figure 1; Table 1; [13],[15]). Weston et al. [13] previously identified a major locus affecting the interspecies male wing size difference, called *wing-size-1* (*ws1*). The *giraulti* allele at this locus (*ws1_g_*) was shown to increase wing size by approximately 60% when introgressed from *N. giraulti* into a *N. vitripennis* background, accounting for 44% of the species difference. To positional clone this major sex-specific wing QTL and to more precisely describe its phenotypic effects we (a) reduced the size of the introgressed sequence flanking the *ws1* locus to a 40kb segment (see fine-scale mapping and cloning below) and (b) backcrossed the introgressed *ws1_g_* segment into a standard *N. vitripennis* strain (AsymCX) genetic background for >10 generations. This strain is referred to as *ws1_g_V*_*40kb*, and is used to more precisely assess the effects of the *ws1_g_* allele on wing size in comparison to the *ws1_v_* allele in the same genetic background. Overall male forewing area of *ws1_g_V_40kb* is 45% larger than the wild-type *ws1_v_V* allele (Figure 1; Table 1; Tukey's HSD test, p<0.001), and the locus accounts for 39% of the species difference in male wing area. Male forewing length and width are similarly increased (Table 1; HSD tests, p<0.001). In contrast, female wing length, width and area are unaffected by the *ws1* allele (Table 1; HSD tests; p>0.05), confirming the sex-specific effects of this locus.

**Table 1:** Basic wing measurements of *ws1* and wild-type strains.

Genotype	Forewing length	Forewing Width	Forewing Area	Head Width	N (Families)
Males (absolute)					
*ws1_v_V*	1065±28	326±11	242000±13000	402±9	40 (8)
Males (relative)					
*ws1_v_V*	1.00±0.03 *a*	1.00±0.03 *a*	1.00±0.05 *a*	1.00±0.02 *a*	40 (8)
*ws1_g_V_40kb*	1.15±0.04 *b*	1.30±0.05 *b*	1.45±0.09 *b*	0.98±0.04 *a*	25 (5)
*ws1_g_G*	1.28±0.04 *c*	1.81±0.05 *c*	2.16±0.13 *c*	1.01±0.03 *a*	40 (8)
Females (absolute)					
*ws1_v_V*	2006±36	913±19	1175000±43000	495±15	40 (8)
Females (relative)					
*ws1_v_V*	1.00±0.02 *a*	1.00±0.02 *a*	1.00±0.04 *a*	1.00±0.03 *a*	40 (8)
*ws1_g_V_40kb*	1.02±0.02 *a*	1.02±0.02 *a*	1.03±0.05 *a*	0.98±0.02 *a*	24 (5)
*ws1_g_G*	0.86±0.02 *b*	0.87±0.02 *b*	0.75±0.03 *b*	0.98±0.02 *a*	40 (8)

Absolute measurements (mean ± standard deviation) for *ws1_v_V* (*N. vitripennis*) males and females are shown (length and width in µm, area in µm^2^). Relative measurements for other genotypes are shown as (mean / *ws1_v_V* mean) ± (standard deviation / *ws1_v_V* mean). *a,b,c*: contrast groups for multiple comparisons (Tukey's HSD) between genotypes within each sex. Groups are unchanged for confidence levels of both alpha = 0.05 and 0.001. N: number of individuals measured, nested in (families).

A more detailed analysis of phenotype was conducted using setae (wing cell hairs) to estimate cell size and cell number effects of *ws1_g_*. Setae have also been used in *Drosophila* to estimate the relative contribution of cell size and cell number to wing size (e.g., [19]). In *Nasonia*, setae cover the distal portion of the wing, but are sparse in the proximal portion (Figure 1). Most of the size increase due to *ws1_g_* is in the distal portion of the wing as well (73% increase distal to the costal cell versus 21% increase proximal). We therefore estimated seta densities in the distal portion of the adult wing after first establishing that there was a relationship between cell number and seta number. Cell density per seta in pupal wings was estimated by DAPI and phalloidin staining (Figure S1). The average number of cells per seta in *N. vitripennis* male forewings is 3.2±0.4 SD, compared to 4.6±0.4 SD in *N. giraulti* (Mann-Whitney U-test, p<0.05, n = 12). In contrast, the *ws1_g_V* introgression shows the same density of cells per seta as *N. vitripennis* (3.2±0.3 SD for each; U-test, p>0.05, n = 12), indicating that this species difference is not under the genetic control of the *ws1* locus. We then estimated cell number by counting total seta numbers on the distal portion of the adult wing and estimated cell size by calculating the distance to each seta's nearest neighbors. Based on these calculations, the *wsl_g_* allele increases overall cell size by 21%±3% (SD) and cell number by 45%±5%, resulting in a 73%±10% increase in area of the distal half of the wing (Table 2; HSD tests, p<0.05).

**Table 2:** Cell number and size effects of *ws1*, as estimated by seta number and area.

Genotype	Seta Number	Seta Area (Nearest 4 Neighbors)	Distal Forewing Area	N (Families)
Males (absolute)				
*ws1_v_V*	580±44	117±2	135000±10000	8 (2)
Males (relative)				
*ws1_v_V*	1.00±0.08	1.00±0.01	1.00±0.07	8 (2)
*ws1_g_V_40kb*	1.49±0.05	1.21±0.03	1.73±0.10	8 (2)

Seta area and seta number are used to estimate cell size and cell number in the distal portion of the forewing. Seta area is the mean area occupied by each seta, based on the distance to each seta's nearest four neighbors. Absolute measurements (mean ± standard deviation) are shown for *ws1_v_V* (*N. vitripennis*) males (all area units in µm^2^). Relative measurements are shown as (mean / *ws1_v_V* mean) ± (standard deviation / *ws1_v_V* mean). N: number of individuals measured, nested in (families).

It would be useful to know whether large or small male wing-size is ancestral in the *Nasonia* lineage. Other closely related species (e.g. *Trichomalopsis sarcophagae*, *T. dubia*, and *Urolepis rufipes*) have males with large functional wings, suggesting that this state is ancestral. However, the situation is complicated by the fact that the most basal diverging *Nasonia* species, *N. vitripennis,* has small wings (Figure 1), whereas the other species form a monophyletic clade [20] that contains both species with intermediate (*N. longicornis*) and large winged males (*N. giraulti* and *N. oneida*) [15],[20]. Thus, we can postulate that either (a) male wing reduction began in the common ancestor to all four species, with subsequent increase in the lineage leading to *N. giraulti* and *N. oneida*, (b) smaller male wing size has independently evolved in *N. vitripennis* and *N. longicornis*, or (c) there has been introgression of one or more small-wing alleles between *N. vitripennis* and *N. longicornis*. Resolution of these alternatives will require more detailed phenotypic and sequence evaluation of the QTL involved in sex specific wing evolution.

### Positional cloning of *ws1*

Positional cloning of the *ws1* locus involved the following steps: (a) recessive lethals flanking *ws1* were generated using already identified linked visible mutants, (b) these were then used to sequentially generate a set of recombinants on both sides of *ws1* for fine-scale mapping and cloning of the gene, (c) a molecular (AFLP) marker tightly linked to *ws1* was identified by genotyping recombinants, (d) this marker was then used to identify a set of BACs covering the region, which were assembled into contigs [21], (e) PCR based markers were developed for determining recombination intervals within the region using sequences from the BAC containing the AFLP marker, end sequences of flanking BACs, and corresponding *vitripennis* and/or *giraulti* markers (Table S1), (f) a set of increasingly finer-scale recombinants were screened to delineate the *ws1* region and finally (g) additional sequence analysis within the cloned region was conducted to identify features within the region and differences between *N. vitripennis* and *N. giraulti*. The latter effort was enhanced by the availability of genome sequences for *N. vitripennis* (Genbank AAZX00000000) and *N. giraulti* (Genbank ADAO00000000) [10] which became available during the course of this project.

**Figure 2:**
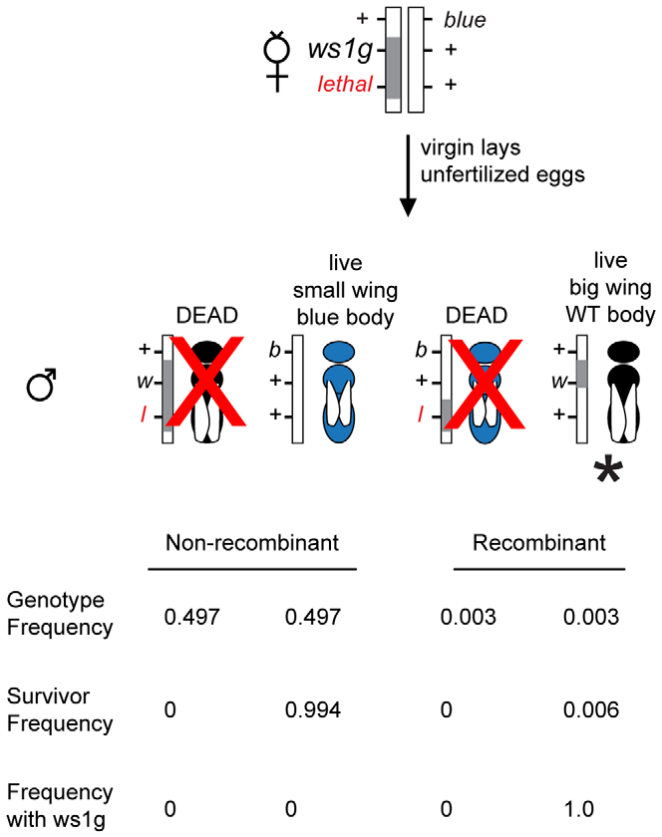
Positional cloning in *Nasonia* using linked lethals. The diagram illustrates a screen for fine-scale recombinants (marked with *) between *ws1_g_* and a lethal located 0.6cM away. Because of the lethal (*l*), the only live haploid males with *ws1_g_* (large wings, *w*) are recombinant. The *blue* (*b*) marker is used as a second phenotypic marker and as a way to identify recombinants on the other side of *ws1.* Gray bar: introgression (*N. giraulti*) sequence. White bar: *N. vitripennis* sequence. Proportions shown are based on estimates of recombination rates between the markers (Figure 3) and assuming no double recombinants.

The method of assembly of BAC contigs is described in [21]. The approach for generating linked lethals and using these for cloning of QTL is described in methods and shown in Figure 2. Due to male haploidy, this method can be used to efficiently screen for recessive lethals linked to any gene of interest within the genome. Briefly, new lethal mutations linked to *ws1* were generated by EMS mutagenesis followed by screening for linkage of the lethal to *ws1*. Use of custom-made lethals in this approach was effective because non-recombinant haploid males with the genotype *lethal ws1_g_* die and, therefore, the only surviving males carrying the *ws1_g_* allele are recombinants between the lethal and wing size locus (+ *ws1_g_*) (Figure 2). These tightly linked lethals increased the “effective” discovery rate of recombinants within the region by 100–200 fold (Figure 2), greatly enhancing efficiency of the positional cloning effort. Thus, we were able to positionally clone *ws1* despite a 10-fold lower recombination rate in this region, 0.10–0.14 centimorgan/megabase (cM/Mb) relative to the genome-wide average of 1.4–1.5 cM/Mb [22].

**Figure 3:**
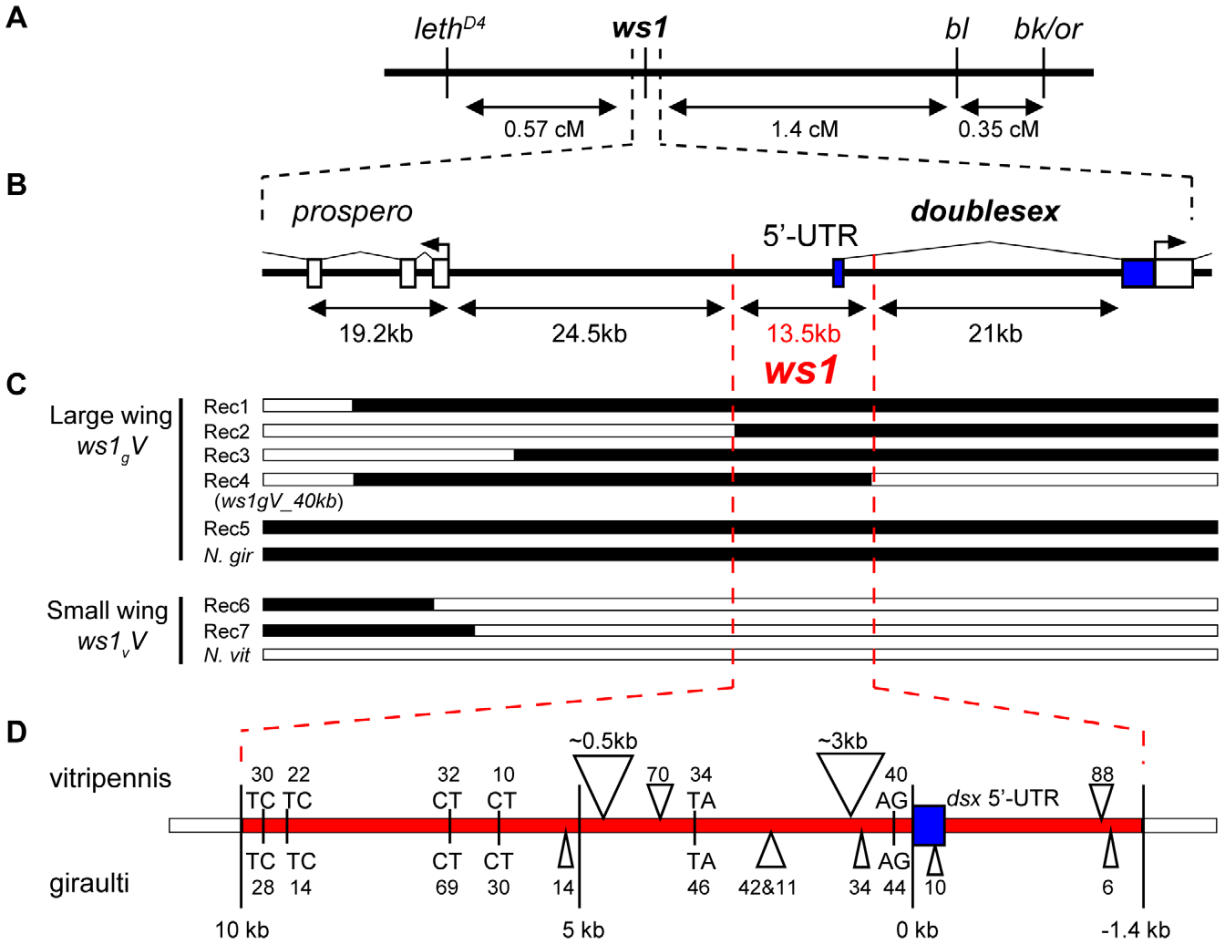
Positional cloning: *ws1* maps to the *doublesex* locus. (A) Recombination map of *ws1* and flanking phenotypic markers used for positional cloning. (B) Genome map of the region around *ws1* including gene annotations. (C) Genotype and phenotype of recombinants near *ws1*. Black: *N. giraulti,* White: *N. vitripennis*. (D) Map of sequence features in the 13.5kb *ws1* locus. Triangles: Insertions, with size given in base pairs (bp). Letters: Microsatellite repeats, with size given in bp. Blue: *dsx* 5′-UTR.

We first isolated the *giraulti* allele of *ws1* by backcrossing into a *vitripennis* background and mapping the locus using visible, lethal and molecular markers (Figure 3A, see also Materials and Methods). Using one line (Rec1, Figure 3C) generated by recombination to the flanking recessive lethal *D4*, we then generated recombinants on the opposite side between *ws1* and the flanking visible marker *bl13*, resulting in a recombinant line showing the *ws1_g_* phenotype with only 40kb of introgressed *giraulti* sequence. This large-winged recombinant strain (*ws1_g_V*_*40kb*) is used for our detailed phenotypic and gene expression analyses. Screening of additional recombinant males further reduced the size of the region known to cause the *ws1_g_* large-wing phenotype to 13.5kb (Figure 3C).

Positional cloning *ws1* shows it to fall adjacent to the protein coding region of *doublesex* (*dsx,* GeneID 100302336), a master sex determination gene found from nematodes to mammals [17],[18]. The 13.5kb *ws1* region contains the *dsx* 5′UTR, promoter and presumed *cis*-regulatory region but notably excludes the protein coding regions of *dsx* or any other gene (Figure 3B). This gene was confirmed as the *Nasonia* ortholog of *doublesex* based on protein domains, expression pattern, sex specific splice forms, and experimental demonstration that splice forms are associated with sex in a gynandromorph producing line [18]. This previous work on *Nasonia dsx* [18] confirmed the coding regions, 5′UTR, 3′UTR, exons and introns for both male and female specific splice-forms (shown in Figure S2) in both *N. vitripennis* and *N. giraulti*.

Upstream of the *ws1* region, the nearest gene was identified by BLAST to be a homolog of the *D. melanogaster* gene *prospero* (*pros,* GeneID 100118692, [18]), 24.5kb away from the *ws1* region (Figure 3B). In *Drosophila*, *prospero* is a transcription factor that specifies cell fate and cell growth in the nervous system [23]. We also identified a single EST (Genbank EV431998) within the 13.5kb region from the *Nasonia* EST dataset [10]. The EV431998 EST contains 5–8 stop codons in each frame and is not spliced, and therefore does not appear to be a protein-coding gene. RT-PCR of EV431998 failed to detect this transcript from wing, leg or whole prepupa cDNA, whereas the primers did amplify from genomic DNA controls. The evidence therefore indicates that the *ws1* phenotype is due to non-coding DNA within the 13.5kb region. 

A number of sequence differences occur between *giraulti* and *vitripennis* in the 13.5kb *ws1* region (Figure 3D), including single nucleotide polymorphisms (SNPs), insertions/deletions (indels) and the insertion of a foldback transposable element into *vitripennis*. We sequenced the full foldback element and found that it does not contain protein coding sequences, but two inverted repeats of approximately 1.5kb and two small “stem loop” regions. Investigation of intraspecific variation in the 13.5kb region and ultra-fine scale mapping is now being undertaken to narrow the region that is involved in the *ws1* phenotype.

### Effects of *ws1* on *dsx* and *pros* expression

**Figure 4:**
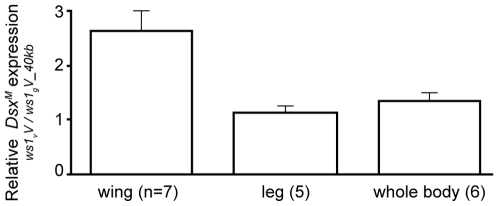
Change in *doublesex* expression due to *ws1.* Expression level change was estimated by quantitative RT–PCR. Note that the *vitripennis dsx* protein-coding region is present in both genotypes (i.e., the *giraulti* region of *ws1_g_V_40kb* does not include the *dsx* coding sequence). Mean expression ratios ± standard errors are shown. Expression ratios greater than 1 indicate higher male *dsx* (*dsx^M^*) transcript level in *ws1_v_V* than in *ws1_g_V_40kb*. Sample size indicates number of independent biological replicates.

Based on our findings revealing *ws1* to contain the 5′ non-coding region of *doublesex*, we next determined whether this region affects *dsx* expression. *Nasonia doublesex* has male and female splice-forms, and experimental evidence supports its role in sex determination in *Nasonia* [18]. As expected, the male splice-form of *ds*x (*dsx^M^*) is present in developing male wings of *vitripennis* (*ws1_v_V*) and *ws1_g_V_40kb* (Figure S3). However, quantitative RT-PCR reveals an estimated 2.4X higher level of *dsx^M^* transcript in developing prepupal male wings of *ws1_v_* relative to *ws1_g_* in the same *vitripennis* genetic background (Figure 4; U-test, p = 0.04, n = 7 biological replicates). In contrast, there is no significant difference between the two genotypes in *dsx^M^* transcript levels in male legs or whole pre-pupae (Figure 4; U-tests, p>0.05, n = 5). We also measured expression of *dsx* in female wings using non-sex-specific primers and observed no clear difference due to *ws1_g_* (median 1.01x expression difference, n = 3; Table 3).

**Table 3:** Gene expression analysis in the *ws1* region.

	Wing	Leg	Whole Prepupa
*dsx^M^ -* Males			
N	7	5	6
Median	2.39	1.07	1.15
Interquartile Range	0.60	0.31	0.35
*pros* - Males			
N	3	3	5
Median	1.07	1.32	1.83
Interquartile Range	0.38	0.65	0.67
*dsx* - Females			
N	3		
Median	1.01		
Interquartile Range	1.57		

Expression level change was estimated by quantitative RT-PCR. Expression ratios greater than 1 indicate higher transcript level in *ws1_v_V* than in *ws1_g_V_40kb*, corrected for control gene (*rp49*) expression. N: number of independent biological replicates. *dsx^M^*: male *dsx* splice form. *dsx* in females used non-sex-specific primers.

No expression difference was found in male wings for the flanking gene *prospero* (Table 3; median 1.07x expression difference between *ws1_v_V* and *ws1_g_V_40kb*). We note that while the introgression appeared to have no effect on *pros* expression in wings, higher *pros* transcript levels were observed in *ws1_v_V_40kb* whole prepupae (Table 3). A likely explanation for this whole-body effect is that the larger 40kb of *giraulti* sequence in the tested strain extends over part of the *pros* gene, possibly affecting its regulation in whole body.

These gene expression data provide a sex- and tissue-specific correlation between *dsx* expression level and wing size. As with other positional cloning studies, our data do not rule out alternative scenarios that could link causative sequence changes in the 13.5kb region to wing size, such as (undetected) effects on *prospero* or long-distance regulation of a different gene. However, we do note that we started with a sex-specific phenotype and “walked” to a region adjacent to a gene known to be involved in somatic sex determination. Future work will be geared towards determining how *ws1* affects sex-specific changes in wing development, and specifically whether changes in *dsx* expression level causally influence male wing size in *Nasonia*.

### Concluding remarks

Our results show that non-coding changes are responsible for the *ws1* male-specific wing phenotype. Unlike studies of candidate genes involved in sex differentiation, the positional cloning approach is candidate-blind, so it is intriguing that the region we identified as causing a sex specific increase in wing size (*ws1*) also contains the 5′ UTR of the sex-signaling gene *doublesex*. Nevertheless a causal relationship between *ws1* and *dsx* has not yet been established. Previous studies [3],[24] have implicated *dsx* in the evolution of sex-specific morphology. But rather than changes in *doublesex* itself, these studies revealed changes in downstream targets of *dsx*, via changes to specific DNA sequences to which DSX protein binds in the *cis*-regulatory regions of the *bric-a-brac* and *desatF* genes and affecting sex differences in abdominal pigmentation and pheromone production. In this study, we observed tissue-specific changes in *dsx* level, possibly due to *cis-*regulation. *Dsx* expression level manipulation has been found to affect cell number of a sex-specific cell type in the *Drosophila* brain [25]. If *dsx* is indeed the mechanism behind *ws1*, it would be the second case of *dsx* regulating sex-specific cell proliferation. Further, it would suggest that sex-specific morphology can evolve by spatially regulated changes in expression within the sex-determining pathway without disrupting other sex-determination functions. Other molecular mechanisms linking the cloned 13.5kb *ws1* region to wing size evolution could also occur, including *cis-*regulation of *prospero,* changes in non-coding RNAs, or long-distance regulation of another gene. *Prospero* is of particular interest because it is a transcription factor known to regulate cell proliferation in the *Drosophila* nervous system [23].

Cell size and cell number regulation are crucial elements of both organ size determination and control of human diseases such as cancer and diabetes [26]. Understanding how growth regulation can evolve therefore has the potential to broaden our knowledge of the operation of these gene networks. One notable example of organ size evolution is *fw2.2,* which regulates tomato fruit size via cell number changes [27]. This gene was positionally cloned [27] and found to be a cell cycle regulator in plants [28]. In *Nasonia*, non-coding *cis-*regulatory evolution at the *ws1* locus causes changes in both cell size and cell number. The two genes flanking the *ws1* region, *doublesex* and *prospero*, have both been found to regulate neuronal cell numbers in *Drosophila*, and *doublesex* does so sex-specifically [23],[25]. How a 45% change in organ size might be achieved by either of these genes, each of which has a conserved homologue in the human genome, remains to be determined.

This study demonstrates the feasibility of positional cloning genes in *Nasonia*. A number of biologically important phenotypic differences occur between *Nasonia* species, which are ripe for genetic investigation using this approach, such as wing and antennal morphology [20], [29]–[30], host preference [14], pheromones and cuticular hydrocarbons [31], diapause [32], hybrid incompatibility [33]–[35], male courtship behavior [36] and female mate preference [20],[37]. The four known *Nasonia* species are all inter-fertile in the laboratory, facilitating the isolation of genes involved in complex trait differences between each species [15],[30]. The availability of genome sequences [10] combined with the haplodiploid positional cloning methods described here now make it possible to determine the evolution of these complex traits on a molecular level in this emerging model organism [9].

## Materials and Methods

### Strains used

Wing measurements were conducted using the inbred *N. vitripennis* strain AsymC and inbred *N. giraulti* strain R16A; these data are also reported in [15]. Gene expression experiments were conducted with the *N. vitripennis* AsymCX strain used for genome sequencing [10], which was derived from AsymC by multiple generations of sib-mating. All wing size and gene expression experiments used the minimal-introgression *ws1_g_V* strain *ws1_g_V_40kb*, produced by backcrossing and selection for recombinants between *ws1* and linked visible and lethal mutants (see Positional Cloning below). This strain contains ∼40Kb of introgressed *giraulti* DNA containing *ws1_g_* in a *vitripennis* genetic background. It was constructed by backcrossing males from minimal-recombinant strain wm114 (Rec 4 in Figure 3C) into AsymCX for 10 generations to produce a homogeneous genetic background. Wild-type *N. vitripennis* and *N. giraulti* are also referred to as *ws1_v_V* and *ws1_g_G* in the text.

### Wing size, cell size, and cell number measurements

Wing measurements were performed as in [15]. Briefly, individual females were given two *Sarcophaga bullata* hosts for 48 hours at 25C after host-feeding for 24 hours on two hosts (which were discarded). Male wing samples were collected from the offspring of single virgin females, while female wing samples were collected from the offspring of single mated females. Adult wings were dissected at the hinge adjoining the thorax and dry mounted on microscope slides under coverslips. Five individuals per family for 5–8 families were mounted; occasional damaged or misshapen wings meant that four individuals per family were measured.

Wings were photographed on a Zeiss AxioImager Z1 compound scope at 10X as mosaic images. Measurements were performed on the wing images using AxioVision 4.6 software (Zeiss). Wing length, width, area, and head width (inter-ocular distance, a measure of body size) were measured as in [15]. Briefly, wing length is the distance between a notch at the proximal anterior end of the costal cell and the distal tip of the forewing. Wing width is the distance perpendicular to the length axis between the most anterior and most posterior points on the wing. Wing area is defined by outlining the wing starting at the proximal anterior notch. Measurements of wild-type *N. vitripennis* and *N. giraulti* shown here (Figure 1) are also reported in [15]. Images of *ws1_g_G* and *ws1_v_V* male wings shown in Figure 1 were cropped to remove other mounted wings which appear in frame but are not related to the displayed image.

Setae, hair-like structures produced by cells on the wing blade, were used to infer changes to cell size and number. This approach has been used to estimate cell size in other insects, particularly *Drosophila* [19]. To determine if seta number is a reasonable estimator of cell number in *Nasonia*, the number of cells per seta was determined at the red-orange-eye pupal stage, where setae are most distinguishable before the wing sclerotizes and cell nuclei disappear. Pupal male forewings were clipped and dissected from the cuticle in 1x TBST (6g Tris, 8.76g NaCl, 1mL Tween, 0.2g NaN_3_, 1L H_2_O, pH 7.5) then fixed on lysine-coated slides in 3.7% formaldehyde. Slides were stained with DAPI and Alexa Fluor488-Phalloidin (Invitrogen, Carlsbad, CA, USA) and mounted in ProLong Gold (Invitrogen). The wing is not completely expanded at this stage and has some three-dimensional structure (Figure S1). Therefore, wings were imaged at 20x as mosaics under multiple focal planes, so that setae on both wing surfaces and all nuclei could be detected. All setae and nuclei were then counted within a 30 µm radius circle placed between the stigma and the distal tip of the forewing.

Cell size and cell number estimates were derived from seta measurements on adult wings. Seta number and area per seta were counted in the distal half of the wing, where setae occur, following [15]. Specifically, a subset of the mounted adult male forewings was re-imaged at 20x through multiple focal planes. Each seta on the dorsal surface of the wing was counted and the area of the seta-containing part of the wing was measured (defined as the area distal to the costal cell, based on the length (proximal-distal) axis described above; [15]). Cell number was inferred from the total seta number. Cell size was inferred by estimating the mean area occupied by each seta based on nearest neighbor distances using a custom perl script. Specifically, the average distance to each seta's four nearest neighbors (*nnd4_i_*) was calculated and then average area per seta across all *i* setae was estimated as the mean of pi * (*nnd4_i_* /2)^2^.

Pairwise comparisons of wing measurements between genotypes (strains) were conducted using Tukey's Honestly Significant Difference (HSD test, [38]), based on ANOVAs using family as a nesting factor within genotype. Because several morphological variables were measured per genotype, we used the conservative Bonferroni correction for multiple tests. P-values shown were corrected by multiplying by the number of tests conducted in each analysis (Table 1: 8 tests (4 variables and 2 sexes per genotype). Table 2: 3 tests (3 variables per genotype).

### Positional cloning methods

Positional cloning efforts were begun by identifying visible mutants linked to *ws1*. Using the original *ws1* introgression from *giraulti* into *vitripennis* (INTw1.1, [13]), it was ascertained that the visible eye color mutant or123 and body color mutant bl13 map near to *ws1*. A second introgression of *ws1_g_* into *vitripennis* containing a large *giraulti* flanking region was used for most of the fine-scale mapping and positional cloning work (strain INT_bkbw, described in [14]). This introgression contains a naturally occurring *giraulti* black eye color allele, *bk_g_,* linked to *ws1*. We found that *bk_g_* fails to complement the *N. vitripennis* mutant *bk576* in heterozygous females. *bk_g_* produces oyster-gray eyes in the *pe333* (peach eye) mutant background, which is easier to see than the black eyes of the mutant in wild-type background. A recombination map of these visible markers is shown in Figure 3A.

To further assist in the positional cloning, recessive lethal mutations linked to *ws1* were generated in the INT_bkbw strain by ethyl methanesulfonate (EMS) treatment of males carrying *ws1_g_*. Ten *bkbw* (*ws1_g_ bk_g_; pe333)* males were placed in 25mm Drosophila vials containing filter paper soaked 10% sucrose solution containing 0.25–0.5% EMS (Sigma Chemical). After 7–10h, males were transferred to a vial containing clean filter paper overnight. Mutagenized males were then crossed to linked visible mutant strain *bl13; pe333*. F1 virgin females were collected, transferred to individual cells of plastic 24-well culture plates (various manufacturers) and given a single fly host to lay eggs. Plates were sealed with a double layer of Micropore tape (product number 1530–03, 3M Corporation) and incubated at 25C. After 48h, females were transferred to a new plate containing a *pe333* male black-stage pupa and a spot of honey water. After mating, the wasps were anaesthetized at 4C and on ice, 2 fly hosts were added to each well, and then incubated at 21C. Newly-created linked recessive lethal markers were identified by distortions in F2 ratios of the visible markers (*ws1_g_, bk_g_* and *bl13*) among the haploid F2 males of the virgin hosting (as in Figure 2) and then linkage relationships were determined. Lethal lines were maintained using heterozygous female offspring. One lethal line, *lethal^D4^*, was primarily used for mapping in this study, due to its close linkage to *ws1* and its position on the side opposite the visible markers (Figure 3A).

To collect recombinant males for positional cloning, females heterozygous for *ws1_g_* and a flanking visible or lethal marker were set as virgins and resulting haploid male progeny were screened for recombination between the marker locus and *ws1* by phenotype (Figure 2). Use of lethals in this approach was especially effective because non-recombinant haploid *lethal ws1_g_* males die; the only surviving males carrying the *ws1_g_* allele were recombinants between the lethal and wing size locus. Penetrance of the lethal genes used was found to be 100%. These tightly linked markers increased the “effective” discovery rate of recombinants within the region by 100–200 fold, greatly enhancing efficiency of the positional cloning efforts.

A crucial step in the cloning effort was identification of molecular markers within the *ws1* region to assist in fine-scale recombinant mapping. Initially this was accomplished by screening the original *ws1_g_V* introgression line and recombinants between it and flanking visible markers for linked Amplified Fragment Length Polymorphism (AFLP) markers. This was done using methods previously reported in [39]. A marker termed “AF1” was identified and found to be tightly linked to *ws1*. The marker was cloned and sequenced. PCR products from primers designed to AF1 were used to screen a BAC library to *N. vitripennis*. Ends of a subset of BACs were sequenced, the library re-screened, and then RFLP typed to assemble a set of contig BACs [21]. BAC-end sequences were then used to generate a set of molecular markers distinguishing *vitripennis* and *giraulti* by PCR and RFLP (Table S1; [21]). Subsequent sequencing of a *giraulti* BAC (Genbank accession AC185330) which included the entire *ws1_g_* region and alignment of *vitripennis* and *giraulti* trace reads from the *Nasonia* genome project were used to identify additional PCR markers for ultra fine-scale mapping, and identification of recombination breakpoints by sequencing. Genotyping primers and PCR conditions are shown in Table S1.

Over 2000 recombinant haploid males were identified between *ws1* and either the visible or lethal marker. These were screened with molecular markers to identify the location of recombination within the region around *ws1*. The most informative recombinants are shown in Figure 3C. These include recombinants produced from the INT_w1.1 introgression (Rec 5) and the INT_bkbw introgression (Recs 1–4, 6–7). To define the *ws1* region further, a strain was established from a recombinant between the flanking *lethal^D4^* and *ws1* that contained a relatively small region of *giraulti* introgression but retained the large wing phenotype (Rec1 in Figure 3C). This strain was used for subsequent recombination to the other flanking region (*bl13* side). A recombinant from this second set containing only 40kb of *giraulti* sequence (Rec4, Figure 3C) yet still showing the “*ws1*” large-wing phenotype (Figure 1) was then backcrossed into the genome-sequenced AsymCX strain for >10 generations, and then purebred. This strain (called *ws1_g_V_40kb*) was used for wing measurements and quantitative PCR. Additional recombinants from these experiments further localized the *ws1_g_* effect to a 10.8kb region in *giraulti*, and a corresponding 13.5kb in *vitripennis* due to insertion/deletion differences. We examined this sequence and flanking regions for gene predictions [10] and also manually scanned the region for open reading frames and ESTs [18]. The region does not contain protein coding sequence for *dsx*, but only the *dsx* 5′ UTR, promoter and *cis-*regulatory region.

### Recombination rate

We estimated the recombination rate between *ws1* and *lethal^D4^* by counting all (living) male offspring of a set of virgin females (*lethal^D4^ ws1_g_*/+ +) hosted for positional cloning. 89 males out of 15594 screened were recombinant (+ *ws1_g_*), a map distance of 0.57cM. Of a larger set of 683 + *ws1_g_* recombinant males screened with the ws1–8 marker (Table S1), 6 were recombinant between ws1–8 and *ws1_g_*, a distance of 36–50kb (uncertainty is due to the uncertainty in the location of *ws1_g_* in the 13.5kb region). Local recombination rate was calculated as [(0.57 cM between *ws1_g_* and *lethal^D4^*) / (683 recombinants between *ws1_g_* and *lethal^D4^*)] *[(6 recombinants between *ws1_g_* and ws1–8) / (36 or 50kb between *ws1_g_* and ws1–8)]  = 0.14 – 0.10 cM/Mb.

### RT–PCR and quantitative RT–PCR

RNA was isolated from wing discs, leg discs and whole individuals at the third instar larvae - prepupal transition. We found that this stage can be precisely identified to a few hours, between defecation of the larvae and ecdysis. Wing and leg discs were dissected from post-defecation prepupae under RNAse free conditions in 1X phosphate-buffered saline. After dissection, tissues were placed immediately on dry ice and if necessary stored at −80°C until RNA was isolated. Independent extractions of tissue were conducted to produce independent biological replicates. Each biological replicate consisted of 15–30 prepupal wings or legs or a single whole prepupa of each genotype (*ws1_v_V* and *ws1_g_V*_40kb). Total RNA was isolated using Trizol (Invitrogen). RNA was then quantified using a Qubit fluorometer (Invitrogen) and a Quant-iT RNA Assay Kit (Invitrogen) or a ND-1000 Spectrophotometer (NanodropTechnologies, Oxfordshire, UK). Expression of EV431998 was tested in RT-PCR with primers TCGAGGCGGATAGTAAGGGC and AACTTTGTATTCCCTCAGCCAC. RT-PCR of other genes (*dsx* and *pros*) and reaction conditions are presented in [18].

For quantitative RT-PCR, first-strand cDNA Synthesis and qPCR were performed using SuperScript™ III Platinum SYBR Green Two-Step qRT-PCR Kit with ROX (Invitrogen) on an Applied Biosystems 7300 Real Time PCR System. RNA samples were split into reverse-transcribed and -RT controls. Male specific *dsx* (*dsx^M^*) was amplified using primers GCGGATGTGGAAGTAGCCAT and AATACTTGAACTTTTGACGATAAGCACT (Figure S2). In females, *dsx* was amplified using non-sex-specific primers CGAGCCACTGCCGAGTAT and TGGTAGCCAAACCGTTGTAAT. *pros* was amplified with GCTGATGTTCTTCTGGGTGAG and CCAGGAAGTTAGGACTCTTGAAG. The ribosomal protein *rp49* was used as a control, with primers CTTCCGCAAAGTCCTTGTTC and AACTCCATGGGCAATTTCTG. All steps were performed according to the respective manufacturer's protocols. Each biological replicate was tested with two primer pairs (e,g., *dsx^M^* and *rp49*). Each experiment was composed of one *ws1_g_V_40kb* and one *ws1_v_V* tissue sample (15–30 wings, 15–30 legs, or one whole prepupa), +RT and –RT, each run in triplicate. The median cycle threshold value of each triplicate was used for calculation. Expression ratios of *dsx* to *rp49* were calculated using the 2^−ΔΔCT^ method [40]: 2̂-((ct[*ws1_v_V dsx^M^*] - ct[*ws1_v_V rp49*]) - (ct[*ws1_g_V_40kb dsx^M^*] - ct[*ws1_g_V_40kb rp49*])). Expression ratios were not corrected for differential amplification efficiency, and so the magnitude of expression ratios should be considered approximate.

## Supporting Information

Figure S1 Wing setae are used to estimate changes in cell size. Example images of seta and nuclear density from male pupal and adult forewings are shown. Adult wing seta numbers and densities were used to infer changes in cell number and cell size due to *ws1* (Table 2), based on estimates of the number of cells (nuclei) per seta in the pupal wing (Data presented in main text). Precise counts were performed by examining multiple focal planes to track setae and nuclei across different depths. The figure shows single focal planes for setae and for nuclei. (A–C) Setae from pupal male forewings. (D–F) Nuclei from pupal male forewings. (G–I) Closeups of seta from adult male forewings (G-I) show changes in seta density (which estimate cell size). All images are from the distal portion of the forewing of *ws1_v_V* (A,D,G), *ws1_g_V* (B,E,H) and *ws1_g_G* (C,F,I). Pupal setae and nuclei images (e.g., A,D) are different fluorescence channels from the same image (coincident, but from different focal planes). Levels were adjusted uniformly for each panel to improve visual contrast. Scale bar for (A–G): 10 μm. Scale bar for (G–I): 10 μm.
(1.78 MB TIF)

Figure S2 Locations of *dsx* primers. Primer locations used for RT–PCR and qPCR of *dsx* are shown in relation to the *dsx* gene model. Male and female splice-forms are adapted from [18]. Lengths are not to scale. Approximate primer locations (half-arrows) are shown on the male splice-form for (a) male-specific *dsx^M^* qPCR, (b) non-sex-specific *dsx* qPCR, and (c) *dsx* RT–PCR.
(0.19 MB TIF)

Figure S3 *Dsx* splicing in male prepupal wings.RT–PCR of the differentially spliced 3’ domain of *dsx* in prepupal male wings is shown. RNA from single male prepupal wings, single male prepupal legs, and single whole pupal females was isolated using the Dynabeads mRNA DIRECT Micro Kit (Invitrogen) by the mini volumes protocol. Black arrowhead: 573bp unspliced (male) product. White arrowhead: 463bp spliced (female) product. Presence of unspliced *dsx* product in females is expected [18]. (A) (i, ii) Standard RT-PCR. Lanes 1,2: *ws1_v_V* male prepupal wing cDNA. Lanes 3,4: *ws1_v_V* male prepupal leg cDNA. Lane 5: *ws1_g_V_40kb* male prepupal wing cDNA. Lane 6: *ws1_g_V_40kb* male prepupal leg cDNA. Lane 7: *ws1_v_V* female whole pupal cDNA included for reference. (Aii) Lane 8: no-template control (image from a separate row of same gel). 1kb: 1 kilobase DNA ladder (Invitrogen). (B) RT-PCR using concentrated cDNA. Specifically, *ws1_g_V_40kb* wing (Lane 9) and leg (Lane 10) cDNAs were re-isolated from the same cDNA preps described above using the poly-T DynaBeads, which were resuspended in PCR mix. Lane 11: female whole pupal cDNA (unconcentrated; same cDNA as Lane 7). Lane 12: no-template control. PCR conditions: dsdsx_FF and dsdsx_FR2 primers (see Figure S2) [18]; 2 min at 94C, 2x (30s at 94C, 30s at 55C, 5 min at 72C), 35x (30s at 94C, 30s at 55C, 45s at 72C), 5 min at 72C.
(0.62 MB TIF)

Table S1 Primers used to genotype recombinants in the *ws1* region. Base pair position in *N. vitripennis* genome assembly v1.0 SCAFFOLD23 is shown. Dashed line denotes markers within the mapped 13.5kb/10.8kb *ws1* region. PCR conditions for all markers: 94C for 2min, 34 cycles of (94C for 30s, 55C for 45s, 72C for 60s), 72C for 10min. Enzyme: Restriction enzyme used to distinguish the amplicons of the two species-genotypes. Assays marked with “indel” did not require enzymes to distinguish the species-genotype; “sequence” requires sequencing of the PCR product.
(0.09 MB DOC)

## References

[1] Kopp A, True JR (2002). Evolution of male sexual characters in the Oriental *Drosophila melanogaster* species group.. Evol Dev.

[2] Kopp A, Duncan I, Carroll SB (2000). Genetic control and evolution of sexually dimorphic characters in *Drosophila*.. Nature.

[3] Williams TM, Selegue JE, Werner T, Gompel N, Kopp A (2008). The regulation and evolution of a genetic switch controlling sexually dimorphic traits in *Drosophila*.. Cell.

[4] Stern DL (2000). Perspective: Evolutionary Developmental Biology and the Problem of Variation.. Evolution.

[5] Carroll SB (2005). Evolution at Two Levels: On Genes and Form.. PLoS Biol.

[6] Hoekstra HE, Coyne JA (2007). The locus of evolution: evo devo and the genetics of adaptation.. Evolution.

[7] Stern DL, Orgogozo V (2008). The loci of evolution: How predictable is genetic evolution?. Evolution.

[8] Wagner GP, Lynch VJ (2008). The gene regulatory logic of transcription factor evolution.. Trends Ecol Evol.

[9] Werren JH, Loehlin DW (2009). *Nasonia*: An Emerging Model System With 10. Haplodiploid Genetics.. CSH Protoc.

[10] Werren JH, Richards S, Desjardins CA, Niehuis O, Gadau J (2010). Functional and evolutionary insights from the genomes of three parasitoid *Nasonia* species.. Science. In press.

[11] Breeuwer JAJ, Werren JH (1990). Microorganisms Associated With Chromosome Destruction and Reproductive Isolation Between Two Insect Species.. Nature.

[12] Bordenstein SR, Werren JH (2007). Bidirectional Incompatibility among divergent *Wolbachia* and incompatibility level differences among closely related *Wolbachia* in *Nasonia*.. Heredity.

[13] Weston RF, Qureshi I, Werren JH (1999). Genetics of a wing size difference between two *Nasonia* species.. J Evol Biol.

[14] Desjardins CA, Perfectti F, Bartos JD, Enders LS, Werren JH (2010). The genetic basis of interspecies host preference differences in the model parasitoid *Nasonia*.. Heredity. In press..

[15] Loehlin DW, Enders LS, Werren JH (2010). Evolution of sex-specific wing shape at the *widerwing* locus in four species of *Nasonia*.. *Heredity*. In press.

[16] Whiting AR (1967). The biology of the parasitic wasp *Mormoniella vitripennis* [ = *Nasonia brevicornis*] (Walker).. Q Rev Biol.

[17] Raymond CS, Murphy MW, O'Sullivan MG, Bardwell VJ, Zarkower D (2000). *Dmrt1*, a gene related to worm and fly sexual regulators, is required for mammalian testis differentiation.. Genes Dev.

[18] Oliveira DCSG, Werren JH, Verhulst EC, Giebel JD, Kamping A (2009). Identification and characterization of the *doublesex* gene of *Nasonia*.. Insect Mol Biol.

[19] Zwaan BJ, Azevedo RBR, James AC, Van 'T Land J, Partridge L (2000). Cellular basis of wing size variation in *Drosophila melanogaster*: a comparison of latitudinal clines on two continents.. Heredity.

[20] Raychoudhury R, Desjardins CA, Buellesbach J, Loehlin DW, Grillenberger BK (2010). Behavioural and Genetic Characteristics of a New Species of *Nasonia*.. Heredity. In press.

[21] Muñoz-Torres M, Saski C, Blackmon B, Romero-Severson J, Tomkins J (2010). Development of bacterial artificial chromosome library resources for parasitoid Hymenoptera (*Nasonia vitripennis* and *Nasonia giraulti*: Pteromalidae).. Insect Mol Biol In press.

[22] Niehuis O, Gibson JD, Rosenberg M, Pannebakker B, Koevoets T (2010). Recombination and its impact on the genome of the haplodiploid parasitoid wasp *Nasonia*.. PLoS ONE. In Press.

[23] Doe CQ, Chu-LaGraff Q, Wright DM, Scott MP (1991). The *prospero* gene specifies cell fates in the *Drosophila* nervous system.. Cell.

[24] Shirangi TR, Dufour HD, Williams TM, Carroll SB (2009). Rapid Evolution of Sex Pheromone-Producing Enzyme Expression in *Drosophila*.. PLoS Biol.

[25] Sanders LE, Arbeitman MA (2008). Doublesex establishes sexual dimorphism in the *Drosophila* central nervous system in an isoform-dependent manner by directing cell number.. Dev Biol.

[26] Kozma SC, Thomas G (2002). Regulation of cell size in growth, development and human disease: PI3K, PKB and S6K.. Bioessays.

[27] Frary A, Nesbitt CT, Frary A, Grandillo S, van der Knaap E (2000). *Fw2.2*: a quantitative trait locus key to the evolution of tomato fruit size.. Science.

[28] Cong B, Tanksley SD (2006). FW2.2 and cell cycle control in developing tomato fruit: a possible example of gene co-option in the evolution of a novel organ.. Plant Mol Biol.

[29] Darling DC, Werren JH (1990). Biosystematics of *Nasonia* (Hymenoptera: Pteromalidae): Two new species reared from birds' nests in North America.. Ann Ent Soc Am.

[30] Gadau J, Page RE, Werren JH (2002). The genetic basis of the interspecific differences in wing size in *Nasonia* (Hymenoptera: Pteromalidae): major quantitative trait loci and epistasis.. Genetics.

[31] Steiner S, Hermann N, Ruther J (2006). Characterization of a female-produced courtship pheromone in the parasitoid *Nasonia vitripennis*.. J Chem Ecol.

[32] Wolschin F, Gadau J (2009). Deciphering proteomic signatures of early diapause in Nasonia.. PLoS ONE.

[33] Breeuwer JAJ, Werren JH (1995). Hybrid Breakdown Between Two Haplodiploid Species: The Role of Nuclear and Cytoplasmic Genes.. Evolution.

[34] Niehuis O, Judson AK, Gadau J (2008). Cytonuclear genic incompatibilities cause increased mortality in male F-2 hybrids of *Nasonia giraulti* and *N. vitripennis*.. Genetics.

[35] Clark ME, O'Hara PF, Chawla A, Werren JH (2010). Behavioural and spermatogenic hybrid male breakdown in *Nasonia*.. Heredity In press..

[36] Beukeboom LW, Van den Assem J (2002). Courtship and mating behaviour of interspecific *Nasonia* hybrids (Hymenoptera, Pteromalidae): A grandfather effect.. Behav Genet.

[37] Velthuis BJ, Yang W, van Opijnen T, Werren JH (2005). Genetics of female mate discrimination of heterospecific males in Nasonia (Hymenoptera, Pteromalidae).. Anim Behav.

[38] Sokal RR, Rohlf FJ (1994). Biometry (3^rd^ edition)..

[39] Vos P, Hogers R, Bleeker M, Reijans M, van de Lee T (1995). AFLP: A new technique for DNA-fingerprinting.. Nucleic Acids Res.

[40] Livak KJ, Schmittgen TD (2001). Analysis of relative gene expression data using real-time quantitative PCR and the 2^−ΔΔCT^ method.. Methods.

