# Supplementary figures and images for: Non-Coding Changes Cause Sex-Specific Wing Size Differences between Closely Related Species of Nasonia
Source: PLoS Genet. 2010 Jan 15;6(1):e1000821. doi: 10.1371/journal.pgen.1000821 (PMC2799512; doi:10.1371/journal.pgen.1000821)

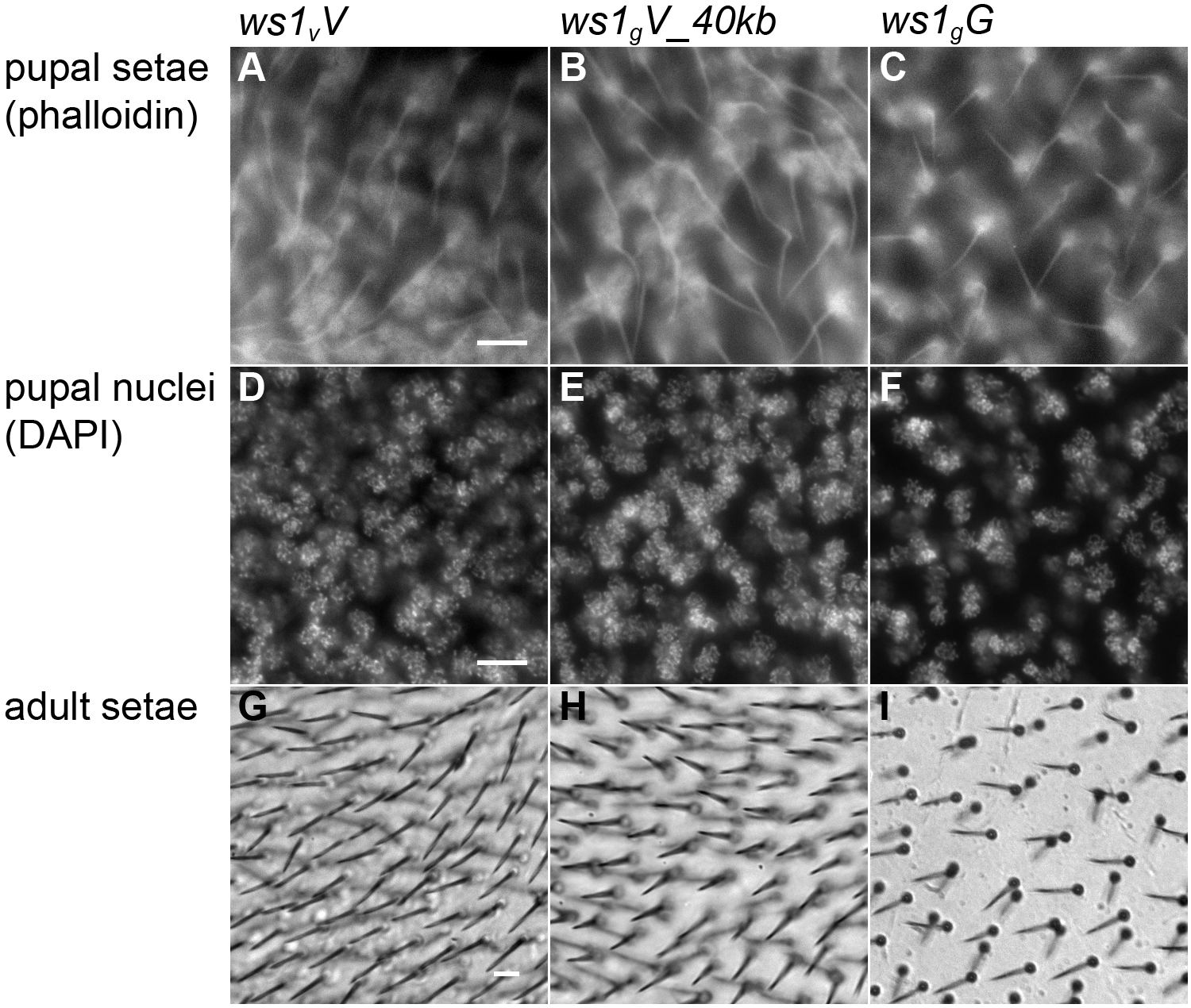

Supplement: Figure S1 — Wing setae are used to estimate changes in cell size. Example images of seta and nuclear density from male pupal and adult forewings are shown. Adult wing seta numbers and densities were used to infer changes in cell number and cell size due to ws1 (Table 2), based on estimates of the number of cells (nuclei) per seta in the pupal wing (Data presented in main text). Precise counts were performed by examining multiple focal planes to track setae and nuclei across different depths. The figure shows single focal planes for setae and for nuclei. (A–C) Setae from pupal male forewings. (D–F) Nuclei from pupal male forewings. (G–I) Closeups of seta from adult male forewings (G-I) show changes in seta density (which estimate cell size). All images are from the distal portion of the forewing of ws1vV (A,D,G), ws1gV (B,E,H) and ws1gG (C,F,I). Pupal setae and nuclei images (e.g., A,D) are different fluorescence channels from the same image (coincident, but from different focal planes). Levels were adjusted uniformly for each panel to improve visual contrast. Scale bar for (A–G): 10 μm. Scale bar for (G–I): 10 μm. (1.78 MB TIF) [file pgen.1000821.s001.tif]

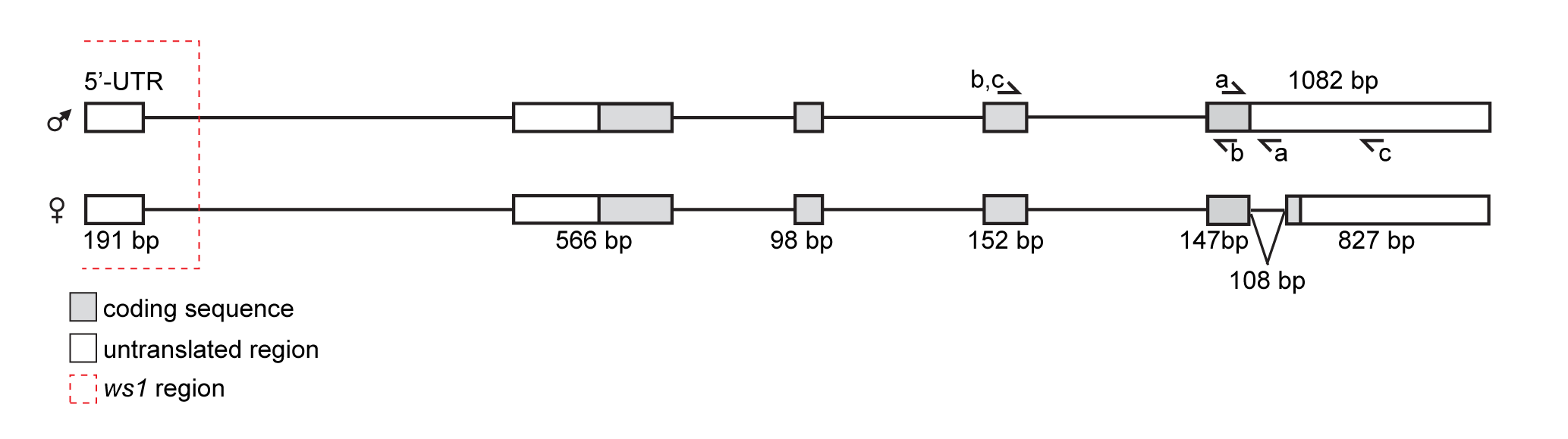

Supplement: Figure S2 — Locations of dsx primers. Primer locations used for RT–PCR and qPCR of dsx are shown in relation to the dsx gene model. Male and female splice-forms are adapted from [18]. Lengths are not to scale. Approximate primer locations (half-arrows) are shown on the male splice-form for (a) male-specific dsxM qPCR, (b) non-sex-specific dsx qPCR, and (c) dsx RT–PCR. (0.19 MB TIF) [file pgen.1000821.s002.tif]

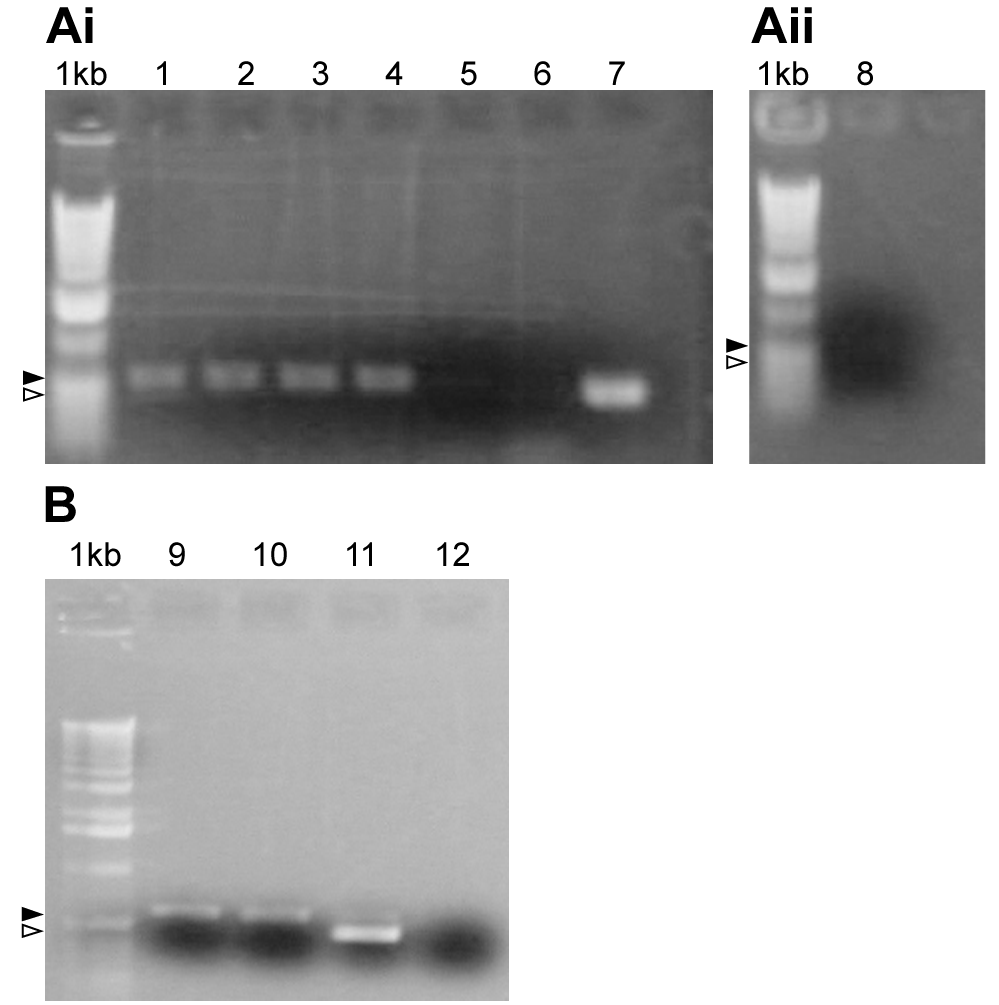

Supplement: Figure S3 — Dsx splicing in male prepupal wings.RT–PCR of the differentially spliced 3’ domain of dsx in prepupal male wings is shown. RNA from single male prepupal wings, single male prepupal legs, and single whole pupal females was isolated using the Dynabeads mRNA DIRECT Micro Kit (Invitrogen) by the mini volumes protocol. Black arrowhead: 573bp unspliced (male) product. White arrowhead: 463bp spliced (female) product. Presence of unspliced dsx product in females is expected [18]. (A) (i, ii) Standard RT-PCR. Lanes 1,2: ws1vV male prepupal wing cDNA. Lanes 3,4: ws1vV male prepupal leg cDNA. Lane 5: ws1gV_40kb male prepupal wing cDNA. Lane 6: ws1gV_40kb male prepupal leg cDNA. Lane 7: ws1vV female whole pupal cDNA included for reference. (Aii) Lane 8: no-template control (image from a separate row of same gel). 1kb: 1 kilobase DNA ladder (Invitrogen). (B) RT-PCR using concentrated cDNA. Specifically, ws1gV_40kb wing (Lane 9) and leg (Lane 10) cDNAs were re-isolated from the same cDNA preps described above using the poly-T DynaBeads, which were resuspended in PCR mix. Lane 11: female whole pupal cDNA (unconcentrated; same cDNA as Lane 7). Lane 12: no-template control. PCR conditions: dsdsx_FF and dsdsx_FR2 primers (see Figure S2) [18]; 2 min at 94C, 2x (30s at 94C, 30s at 55C, 5 min at 72C), 35x (30s at 94C, 30s at 55C, 45s at 72C), 5 min at 72C. (0.62 MB TIF) [file pgen.1000821.s003.tif]
