# Supplementary material for: Non-Coding Changes Cause Sex-Specific Wing Size Differences between Closely Related Species of Nasonia
Source: PLoS Genet. 2010 Jan 15;6(1):e1000821. doi: 10.1371/journal.pgen.1000821 (PMC2799512; doi:10.1371/journal.pgen.1000821)
Supplement: Table S1 — Primers used to genotype recombinants in the ws1 region. Base pair position in N. vitripennis genome assembly v1.0 SCAFFOLD23 is shown. Dashed line denotes markers within the mapped 13.5kb/10.8kb ws1 region. PCR conditions for all markers: 94C for 2min, 34 cycles of (94C for 30s, 55C for 45s, 72C for 60s), 72C for 10min. Enzyme: Restriction enzyme used to distinguish the amplicons of the two species-genotypes. Assays marked with “indel” did not require enzymes to distinguish the species-genotype; “sequence” requires sequencing of the PCR product. (0.09 MB DOC) [file pgen.1000821.s004.doc]

| Primer Name | Forward Primer | Reverse Primer | Difference or Restriction Enzyme | Position |
| --- | --- | --- | --- | --- |
| 67D12.gg | AGCTTGATGCAGCGTGACTTC | AAATGTGCGACGAGCTGAGC | indel | 60719 |
| 01N08.gg | GCGAAAGCGAGCCGATTAAG | GTGAGATTGCTCAGCGGTATCAAG | vit only | 139249 |
| AF1 | GTCGCACCACTTGCTACAGA | GAATTGGCCAACCTTCATTG | Dde I | 141421 |
| Ws1-5 | CGAGCGAGAAGACTATGAAACC | ATTAGCCTATTCGAAGAGAGACG | indel | 152819 |
| Ws1-20 | AGCTGTCTCAGCTCTCTTCGTC | TGCAGTGTAAGTCGTTTGCTTC | sequence | 154085 |
| Ws1-22 | CGTGTGGGAATAGAGCTTTG | CTCATCCGCATTGCGAC | indel | 159827 |
| Ws1-mRNA | TTACTCGTTCGACATGAAGATTG | TACTACCGTTTCATGAACCCAG | Mbo I | 171211 |
| Ws1-13 | AACGGAAATCGGATCAAGC | TCTTTCTCTCACACTCGCACAC | Apo I | 172405 |
| Ws1-14 | CATACTAGATATGCGTGCATCAAG | ATTTAACGGCACGAAAAAGCAATGG | sequence | 174009 |
| Ws1- 7 | GCTGTTGTTATTGCTCAAACTACG | AAGCAGTGTATTGAAGTTACACCAG | sequence | 175822 |
| Ws1-16 | TAGCCTTTCGCATTAGCCTAC | GCACTACGCAAATTACAATATTCC | indel | 179692 |
| 41M16.bb | GTTAATGAACGGGTTGTCGTG | ACCGACTGTACCAAGATTCCTAG | indel | 181519 |
| Ws1-31 | AAGCCGTAAGAGCTCCTTAGAG | ACGAAGAATTTGTATGAAATCGAC | indel | 188849 |
| Ws1-32 | ATAAACGCGATAATATACACACCTC | TCTTGATCGGCAGACGGTC | indel | 193964 |
| Ws1-33 | CTCGCGTTGCTTAATCTTATCTG | TACTACTTCGTCGCTATTTTCTGAG | sequence | 195658 |
| Ws1-35 | GAATATTCTCCAAGATTGCGAAAC | TAAGAAACTGATTGGCACGTAAAC | sequence | 196739 |
| Ws1-34 | TGCCGAATTTATCGTTACAAG | CTTTTACCATATTCAAATAAGGAGC | sequence | 196946 |
| Ws1-0 | YGAGACGACGAGTAAATCAGTAGC | TGACTCTTCTGTTTTTCACTCTCC | indel | 197881 |
| Ws1-25 | CGAATCTCTTCGTCCAGCTATG | ATGTACACACGACATTCACGG | indel | 206793 |
| Ws1-43 | ATCGCATCCTCGCAGTGG | GATCTCGTTTGACTCTCGTTACC | Alu I | 207516 |
| Ws1-44 | TTATCATAGCTGTGCCGTCG | TCATTGCGTGATTATCATTGC | Mbo II | 208251 |
| Ws1-46 | CAGTGAGGCATCGTGACCA | CTGAGCGTAGGTGTATTGATGTGT | Ase I | 208840 |
| Ws1-45 | GCTTAAATCTTATCGTAGGTATCG | GTTATGATCCATATTGTACGTGTGC | indel | 209639 |
| Ws1-24 | CTCACACAAGCATGTACTTGCTC | CTAACATGACACAAGTTTTGAGTTAC | indel | 210507 |
| Ws1-26 | AACGTGAACAAAGTTTTACAATCTG | CGACAACTACATACTACTACCTACCG | Rsa I | 213775 |
| Ws1-39 | CTCTCGATGTCTTTGCGTGG | GGCGACAGGTGCATAAGG | Dde I | 215089 |
| Ws1-40 | TCGAACTGAGGACTGGTGTAGC | GAATTGTGGGAATGACTTTACG | Mse I | 216237 |
| Ws1-41 | GCTGCAAACGAACAGAGG | TCGATCACATGACCACAACG | Acc I | 217584 |
| Ws1-36 | TGATCTGTTTGATATGTAGTAGTCGC | ATTGAATGTCTGTAGACTACGGAGC | Rsa I | 217787 |
| Ws1-42 | TAGACCTATTTCGATAGAGTCACCC | GATTTGCTTCCAAGTTCTCTCG | Mnl I | 219043 |
| Ws1-27B | CTTCTAGATTTTCTCGATTCGTC | GATTCGGTATTTATAATAGCCTGC | Ban II | 220194 |
| Ws1-37 | GGTCCATACAAAGAGGAAGTCG | AAGACTCAAAGCAAGCAGTTCG | Rsa I | 223686 |
| Ws1-38 | CGTAATGGAGATTATCACACAGG | GGACATTAAATCTTAGTGAAAACACG | Bsa I | 226451 |
| Ws1- 3 | TCGTTACAGGGAGTTCTACTACATC | CCAGGAAGTTAGGACTCTTGAAG | Mfe I | 234561 |
| Ws1- 10 | CGGACTCGCAGCTATCTTTC | CGTTATACACACAACGATCCGA | Ava I | 238893 |
| Ws1-11 | AGTCTTTCTCTCTGCATACGACC | CGGAAATCTCTCGCCTCTG | sequence | 240390 |
| Ws1-17 | GCTCGAAGATCGAAAGGGTC | AGGAGGGCTGCTTCTTTGTC | sequence | 240953 |
| Ws1-18 | CATAATGTTTTCATTCGATCATCTC | GCTACGCAGTGTATTTACAGCTTC | Rsa I | 241431 |
| Ws1-12 | CTGACGAGAGCTAAAAGTTATACG | TTCGCTCGCCCGCTAC | sequence | 242025 |
| Ws1- 8 | TTGCGTTAGCCCGATAAACTC | GAGACTCTGAATTGATTTCGTTTG | indel | 243835 |
| Ws1- 9 | GAAAAAGAGGCTTATATTCTTTGC | TAACCAAATTGACGACGGAAC | Nla IV | 248624 |
| 64F12.bb | TAGAGTCGACCTGCAGGCATG | GGACCTAAAGACCGAGCTCAAG | Aci I | 253108 |
| 01N08.bb | GGCGAACTGCATACATGCGC | TCTGAGCAGCTAATAAAGGCAGAGC | Hae II | 283175 |
| 19G07.sp | CAGTGATTTTGCACTCGTTC | TTACCTTTACGGCTTGTTTG | indel | 517271 |
